# Canonical Stimulation of the NLRP3 Inflammasome by Fungal Antigens Links Innate and Adaptive B-Lymphocyte Responses by Modulating IL-1β and IgM Production

**DOI:** 10.3389/fimmu.2017.01504

**Published:** 2017-11-09

**Authors:** Mohamed F. Ali, Harika Dasari, Virginia P. Van Keulen, Eva M. Carmona

**Affiliations:** ^1^The Thoracic Diseases Research Unit and the Division of Pulmonary and Critical Care, Department of Medicine Mayo Clinic and Foundation, Rochester, MN, United States

**Keywords:** β-glucan, B-lymphocytes, inflammasome, fungi, CpG, NLRP3, IL-1β, IgM

## Abstract

The NLRP3 inflammasome is activated in response to different bacterial, viral, and fungal pathogens and serves as modulator of different pattern recognition receptors signaling pathways. One of the main functions of NLRP3 is to participate in IL-1β maturation which is important in the host defense against *Pneumocystis* and other fungal infections. However, dysregulation of NLRP3 and IL-1β secretion are also implicated in the pathophysiology of many auto-inflammatory disorders. Often time’s inflammatory flares are preceded by infectious illnesses questioning the role of infection in autoimmune exacerbations. However, we still do not fully understand the exact role that infection or even colonization plays as a trigger of inflammation. Herein, we investigated the role of NLRP3 in circulating B-lymphocytes following activation with two major microbial antigens (β-glucan and CpG). NLRP3 was determined essential in two independent B-lymphocytes processes: pro-inflammatory cytokine secretion and antibody regulation. Our results show that the β-glucan fungal cell wall carbohydrate stimulated B-lymphocytes to secrete IL-1β in a process partially mediated by Dectin-1 activation *via* SYK and the transcription factors NF-κB and AP-1. This IL-1β secretion was regulated by the NLRP3 inflammasome and was dependent on potassium efflux and Caspase-1. Interestingly, B-lymphocytes activated by unmethylated CpG motifs, found in bacterial and fungal DNA, failed to induce IL-1β. However, B-lymphocyte stimulation by CpG resulted in NLRP3 and Caspase-1 activation and the production and secretion of IgM antibodies. Furthermore, CpG-stimulated IgM secretion, unlike β-glucan-mediated IL-1β production, was mediated by the mammalian target of rapamycin (mTOR). Inhibition of NLRP3 and the mTOR pathway in CpG activated B-lymphocytes resulted in impaired IgM secretion suggesting their participation in antibody regulation. In conclusion, this study describes a differential response of NLRP3 to β-glucan and CpG antigens and identifies the NLRP3 inflammasome of human circulating B-lymphocytes as a modulator of the innate and adaptive immune systems.

## Introduction

The host immune system greatly determines the severity of fungal diseases. In patients with an intact immune system, fungal infections are often clinically asymptomatic or manifest as a mild respiratory illness. In the immunocompromised host, however, fungal infections can disseminate and result in a life-threatening event with high morbidity and mortality. Fungal diseases are on the rise, likely as a result of increasing use of immunosuppressive agents to treat malignancies and autoimmune diseases. Better understanding of fungal immunity will help with the development of alternative antifungal therapeutic strategies that enhance specific aspects of host immunity.

B-lymphocytes, well-known players of the adaptive immune response, react to fungal pathogens by generating antibodies and by releasing inflammatory cytokines (1, 2). In the presence of T cells, B-lymphocyte responses are characterized by isotype class switch and generation of memory and long-lived plasma cells leading to the production of high affinity immunoglobulins (Igs) mostly of the IgG subtype. In the absence of T cells, B-lymphocytes still generate Igs but these are of low affinity and mostly IgM. T-cell independent activation of B-lymphocytes also results in the release of a variety of cytokines and chemokines which are mostly triggered by the activation of pattern recognition receptors (PRRs) such as toll-like receptors (TLRs) and C-lectin receptors (3). PRRs are expressed by most innate immune effector cells, including B-lymphocytes and play a critical role in the detection of pathogens by recognizing conserved pathogen-associated molecular patterns like β-glucan and CpG. β-glucans are highly immunogenic carbohydrates found in the cell wall of many fungi including *Aspergillus* spp., *Candida* spp., and *Pneumocystis* while CpG are highly immunoreactive unmethylated motifs found in bacterial and fungal DNA (4, 5). While each signal using specific PRRs, both have potent immunomodulatory properties and can activate B-lymphocytes directly without the participation of T cells. B-lymphocyte activation by β-glucan and CpG results in the secretion of a specific profile of pro-inflammatory cytokines and chemokines important for the orchestration and activation of monocytes, macrophages, and neutrophils and therefore essential for host defense against fungal and other infections (6–8).

The NLRP3 inflammasome is generally triggered by infection or tissue damage and participates in the processing of mature and bioactive IL-1β from its precursor and inactive form (pro-IL-1β) (9, 10). Since increased production of IL-1β is known to be important for the clearance of fungal infections and little is known about the contribution of B-lymphocytes to the innate immune fungal defense, we sought to investigate the role of NLRP3 activation in B-lymphocytes upon fungal β-glucan stimulation and compare it with B-lymphocyte responses to CpG.

The assembly of the inflammasome classically involves the recruitment of a Nod-like receptor (NLR), an adaptor protein (ASC) and a protease (pro-caspase-1). Depending on the stimuli, the activation of NLRP3 can follow a canonical pathway that involves caspase-1 activation or a non-canonical pathway that is independent of caspase-1. In the particular case of β-glucans, the data are contradictory and both pathways have been described (11–13). In B-lymphocytes, however, the participation of NLRP3 in cytokine regulation and other processes has not been well characterized.

Herein, we describe a dual function of the NLRP3 inflammasome in activated peripheral human B-lymphocytes as a modulator of IL-1β secretion as well as antibody production. While fungal β-glucans andCpG were both able to elicit activation of the NLRP3 inflammasome, the mechanisms of activation were divergent with β-glucan inducing IL-1β secretion and CpG an increase of IgM. Furthermore, CpG activation of NLRP3 was dependent on the activation of the mammalian target of rapamycin (mTOR) pathway while β-glucan activation was mTOR-independent.

## Materials and Methods

### Reagents and Antibodies

Endotoxin-free buffers and reagents were scrupulously used in all experiments. Curdlan, Zymosan, and Laminarin were purchased from Sigma Chemical Co. (St. Louis, MO, USA). *Aspergillus fumigatus* β-glucan preparations were isolated as previously described (14). To ensure that all glucan preparations were free of endotoxin prior to use in culture, Curdlan, Zymosan, and *A. fumigatus* glucans were vigorously washed 10 times with distilled physiological saline, incubated rotating overnight with polymyxin B (Sigma, St. Louis, MO, USA) at 4°C, then vigorously washed again with distilled physiological saline. The final preparations were assayed for endotoxin with the limulus amebocyte lysate method using Pyrosate Rapid Endotoxin Detection Kit (Associates of Cape Cod, East Falmouth, MA, USA) and found to consistently contain less than 0.25 EU/ml. Glucans were pulse sonicated 10 times using a Branson digital sonifier (VWR Scientific, Radnor, PA, USA) at 35% amplitude immediately before addition to the cultures. The Erk 1/2 inhibitor (PD98059), SYK inhibitors (Piceatannol and R406), and NF-κB inhibitor (Bay11-7085) were all obtained from Calbiochem, Inc. (San Diego, CA, USA). AP-1 inhibitor (SR 11302) was from R&D Systems, Inc. (Minneapolis, MN, USA). Caspase inhibitors (Ac-YVAD-CMK and Ac-YVAD-CHO) were purchased from Cayman Chemical (Ann Arbor, MI, USA), VX-765 was from AdooQ Bioscience (Irvine, CA, USA), and Rapamycin was purchased from Selleck Chemicals (Houston, TX, USA). KCl and Adenosine 5′-triphosphate (ATP) were from Sigma and oxidized ATP (oxATP) and MCC950 were from EMD Millipore (Billerica, MA, USA). Phosphorothioate-protected CpG oligonucleotide (5′-TCGTCGTTTTGTCGTTTTGTCGTT-3′) ODN 2006 and *A. fumigatus* oligonucleotides; AF1 (5′-TCGTCGTTGTCGTCGTC-3′) and AF2 (5′-TCGTCGTTGTCGTC-3′) were commercially synthesized by Integrated DNA Technologies, Inc. For most of the experiments, we used CpG ODN 2006 (CpG) unless otherwise specified. Antibodies recognizing the inflammasome components caspase-1 and IL-1β were purchased from Santa Cruz Inc. (Dallas, TX, USA), while NLRP3, ASC, and the mTOR pathway antibodies; mTOR, phospho-mTOR, S6K, and phospho-S6K were from Cell Signaling Technology, Inc. (Danvers, MA, USA). The neutralizing antibody for Dectin-1 was purchased from AbD Serotec (Raleigh, NC, USA) and Isotype control antibody was from R&D Systems, Inc. (Minneapolis, MN, USA). All other reagents were obtained from Sigma-Aldrich (St. Louis, MO, USA) unless specified otherwise.

### Leukocyte Isolation and Culture

All methods were carried out in accordance with relevant guidelines and regulations. Human B-lymphocytes were isolated as previously described (7). Briefly, B-lymphocytes were isolated from acid citrate dextrose anticoagulated blood obtained from de-identified healthy volunteer platelet donors in accordance with the current regulations by the AABB and the US Food and Drug Administration (15) and by the Mayo Clinic Institutional Review Board (IRB). Ethics committee approval was not required according to the local and national and guidelines. Cells were isolated using RossetteSep B-cell enrichment cocktail according to the manufacturer’s protocol (StemCell Technologies, Vancouver, BC, Canada). The enriched B-lymphocyte population was repeatedly observed to contain an average of 93.1% ± 2.3% B-lymphocytes. Sorting of human CD19+ B-lymphocytes into naïve (CD27−) and memory (CD27+) B-lymphocytes was performed using mouse anti-human CD27-APC-Vio770 and mouse anti-human CD19-PE antibodies from Miltenyi Biotec (San Diego, CA, USA), on a FACSAria II SORP flow cytometer running FACSDiva v 6.1.3 software.

### RNA Isolation and Real-time qPCR Analysis

Total cellular RNA was isolated from B-lymphocytes using RNeasy Plus Universal Mini Kit (Qiagen) according to the manufacturer’s instructions. For cDNA synthesis, total RNA concentrations and purity were determined using a Nanodrop ND-1000 spectrophotometer, and 1 µg RNA was used in a 20 µl reaction mixture using a Verso cDNA Synthesis Kit (Thermo Scientific). Quantitative real-time PCR was performed in 10 µl reaction in a 96-well plate using 2 µl of diluted cDNA with SYBR Premix Ex Taq (Clontech Laboratories, Inc.) on a ViiA7 Real-Time PCR Detection System (Life Technologies). The data were analyzed with ViiA7 software, version 1.2.4 (Life Technologies). Relative transcript expression of IL-1β was determined using the comparative Ct method, and was normalized to GAPDH transcripts of the same cDNA samples. The results were expressed as % of the target gene relative to that of GAPDH and plotted as the mean ± SEM. Experimental reproducibility was confirmed using three biological replicates from independent experiments which used B-lymphocytes from three different donors. The primers used for amplification were: IL-1β, 5′-ATGCACCTGTACGATCACTG-3′ and 5′-ACAAAGGACATGGAGAACACC-3′; GAPDH, 5′-ACATCGCTCAGACACCATG-3′ and 5′-TGTAGTTGAGGTCAATGAAGGG-3′.

### Cytokines and IgM Detection

B-lymphocytes (4 × 10^5^ cells/well in 96-well plates) were cultured with 1 µg/ml of CpG (ODN 2006), or with 1 µg/ml AF1, 1 µg/ml AF2, 200 µg/ml of Curdlan (Curd), 200 µg/ml Zymosan (Zym), 200 µg/ml or 200 µg/ml*A. fumigatus* β-glucan (AspG) in culture medium for 24 h for IL-1β and other cytokines and 5 days for IgM unless otherwise indicated. Cell supernatants were then analyzed for IL-1β, TNFα, IL-6, MMP7, and IgM production using human DuoSet ELISA kit for MMP-7 from R&D Systems, Inc. (Minneapolis, MN, USA), BD OptEIA human ELISA kit for IL-1β and Ready-Set-Go ELISA kit for TNFα, IL-6, and IgM from Thermo Fisher (Rochester, NY, USA). ELISAs were performed according to each manufacturer’s instructions. Inhibitors [50 and 100 µM Caspase-1 inhibitors (VX-765 and YVAD.CMK), 50 mM KCl, 50 µM oxATP, 100 µM MCC950, 40 µM Piceatannol, 5 µM R406, 10 µM Bay11-7085, 10 µM SR11302 or 1 mg/ml Laminarin] and neutralizing antibodies (5 µg/ml anti-Dectin-1 IgG or mouse IgG) were incubated with B-lymphocytes for 1 h prior to addition of CpG or β-glucan preparations. Inhibitors were dissolved in dimethyl sulfoxide unless otherwise indicated. Solvents were added to all treatments to assure that solvents concentrations are the same in all treatment conditions.

### Caspase-1 Activity Assay

Caspase-1 activity was determined by a Caspase-1 Fluorometric Assay (BioVision, Mountain View, CA, USA), according to the manufacturer’s instruction. Briefly, 10^7^ B-lymphocytes were stimulated with Curdlan and CpG in the presence of the caspase inhibitor YVAD-CMK, incubated at 37°C for 24 h and lysed with cell lysis buffer on ice for 10 min. Then, equal volumes of 2× reaction buffer and YVAD-AFC were added to the 200 µg of lysates in a 96-well plate and incubated for 2 h at 37°C. The samples were read on a Softmax microplate reader (Molecular Devices, Sunnyvale, CA, USA) with a 400 nm excitation filter and 505 nm emission filter. The caspase-1 activity is expressed relative to the unstimulated control.

### Cellular Viability

Cell viability was assessed using the XTT Cell Proliferation Kit II (Roche Molecular Biochemicals, Indianapolis, IN, USA) according to the manufacturer’s protocol. This assay measures the conversion of sodium-3′-[1-(phenylaminocarbonyl)-3,4-tetrazolium]-bis(4-methoxy-6-nitro) benzenesulfonic acid hydrate (XTT) to a formazan dye through electron coupling in metabolically active mitochondria using the coupling reagent *N*-methyldibenzopyrazine methyl sulfate. Only metabolically active cells are capable of mediating this reaction, which is detected by absorbance of the dye at 450–500 nm. Briefly, 50 µl of the XTT labeling mixture was added to the 100 µl of growth medium containing B-lymphocytes and different concentrations of the inhibitors. The XTT labeling mixture was added in parallel samples 24 h after the addition of the various inhibitors. A set of blanks was also included that did not contain cells and was treated identically as the normal samples. In addition, a set of solvent controls was also included for each inhibitor. Absorbance was measured at 6 h after XTT addition. All treatments were performed in three replicates. Only inhibitor concentrations that elicited less than 20% net toxicity were used in these assays.

### Preparation of Cell Lysates, Electrophoresis, and Immunoblotting

Total cellular proteins were obtained from B-lymphocytes following the described culture conditions. Briefly, the cells were washed with cold PBS twice and lysed in RIPA buffer (50 mM Tris–HCl pH 7.4, 15 mM NaCl, 0.25% deoxycholic acid, 1% NP-40) freshly supplemented with 1 mM phenylmethylsulfonyl fluoride, mammalian protease inhibitor mixture, 10 mM Na Fluoride, and 1 mM Na Orthovanadate. Cells were kept on ice for 15 min and then the lysates were centrifuged at 12,000 × *g* for 10 min at 4°C. The resultant soluble supernatant contained total cellular protein. Protein concentrations were determined with the Bio-Rad protein assay (Hercules, CA, USA) using BSA as the standard. Equal amounts of total cellular proteins were separated on SDS-10% polyacrylamide gels with Precision Plus Protein Dual Color Standards (Bio-Rad; Hercules, CA, USA) being used as the molecular weight standards. Proteins were transferred to Immobilon-P membranes (Millipore, Bedford, MA, USA). Membranes were then blocked at room temperature for 30 min with 1% BSA/TBST (TBS, pH 7.4, 1% BSA, 0.1% Tween 20) and incubated overnight at 4°C in a blocking solution containing primary antibodies at the appropriate dilutions. After washing with TBST, the membranes were incubated with horseradish peroxidase-conjugated secondary antibodies for 1 h. Immunoreactive bands were detected with SuperSignal West PicoChemiluminescent Substrate from Thermo Scientific (Rockford, IL, USA). Actin was used as loading control for the cell lysates and a non-specific protein for the cell supernatant.

### Statistical Analyses

All data are presented as the mean ± SEM, from at least three independent experiments from different biological donors, unless otherwise specified. An experimental run included at least three different donors. The data were first analyzed using one-way ANOVA, the differences between the individual groups were compared using multiple comparisons post-test unless otherwise indicated. Statistical analysis was performed using GraphPad Prism Version 5 (GraphPad Software, La Jolla, CA, USA).

## Results

### IL-1β Is Induced by Fungal β-Glucans in a T-Cell Independent Manner

**Figure 1:**
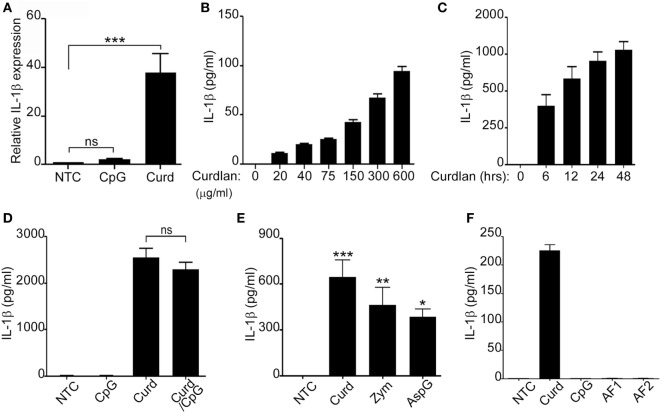
Fungal β-glucans elicit IL-1β secretion in B-lymphocytes. Quantitative real-time PCR for IL-1β mRNA was determined in non-treated cells (NTC) or B-cells stimulated with CpG or Curdlan (Curd). Expression was normalized to GAPDH **(A)**. IL-1β levels were measured by ELISA in the cell supernatant of curdlan stimulated cells at different concentrations **(B)**, for different periods of time **(C)**, with CpG and Curdlan as indicated **(D)** with different β-glucan preparations: Curdlan, Zymosan (Zym), and *Aspergillus fumigatus* (AspG) **(E)** and with different CpG motifs from *A. fumigatus*: AF1 and AF2 **(F)**. Stimulation was done overnight unless otherwise indicated. 200 µg/ml β-glucan and 1 µg/ml CpG, AF1, and AF2 were used unless otherwise specified. Data are representative of at least three donors in each independent experiment (*n* = 3). *p < 0.05, **p < 0.01, ***p < 0.0001, ns, not significant (*p* > 0.05).

B-lymphocytes respond to fungal β-glucans and bacterial DNA in a T-cell independent manner by releasing cytokines and chemokines important for the acute inflammatory response (8, 16). We have previously shown that β-glucan-stimulated peripheral human B-lymphocytes are a source of IL-8, IL-6, and TNFα (7). Additionally, supernatant from β-glucan-stimulated B-lymphocytes was able to significantly increase neutrophil chemotaxis suggesting the contribution of B-lymphocytes in the acute inflammatory process (7). To further understand the role of B-lymphocytes in fungal defense and since IL-1β has been shown to participate in fungal clearance and neutrophil recruitment (17, 18), freshly isolated peripheral B-lymphocytes from normal donors were stimulated with either β-glucan (Curdlan) or CpG (CpG ODN 2006) and assessed for IL-1β expression. RNA levels of IL-1β were significantly elevated in β-glucan-stimulated cells but not in those stimulated with CpG (Figure 1A). Protein levels of IL-1β were next confirmed in the cell supernatants by ELISA. As expected, β-glucan-stimulated cells secreted significant amounts of active IL-1β in a dose and time responsive manner (Figures 1B,C). The addition of β-glucan to CpG did not enhance IL-1β stimulation (Figure 1D). Furthermore, IL-1β secretion was not restricted to curdlan β-glucans as other β-glucan preparation such as zymosan and β-glucan from *Aspergillus* were also potent inducers of IL-1β (Figure 1E). Similarly, failure to induce IL-1β secretion was not restricted to CpG ODN 2006, since two different CpG motifs present in *A. fumigatus* DNA (AF1 and AF2) (4) also failed to stimulate IL-1β secretion (Figure 1F). The differential response of B-lymphocytes to β-glucan and CpG was intriguing, though similarly observed by our group in the setting of IL-8 and IgM (7). Our published studies demonstrated that β-glucan-induced IL-8 secretion had no effect on IgM while CpG stimulated IgM secretion did not participate in IL-8 secretion (7).

### β-Glucan and CpG Induce Activation of the NLRP3 Inflammasome in Human Circulating B-Lymphocytes

**Figure 2:**
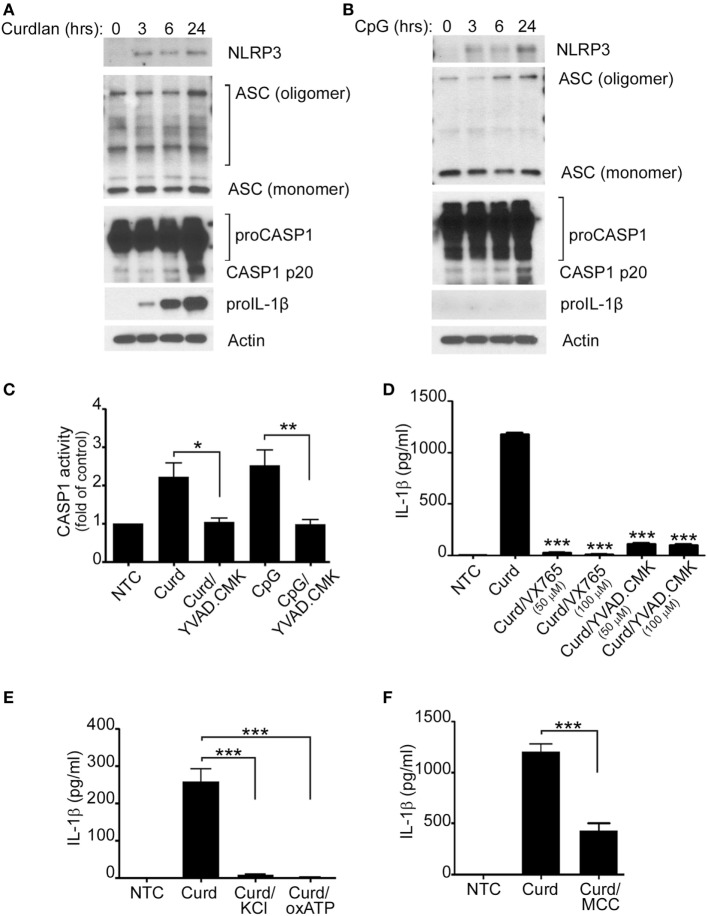
IL-1β secretion requires the NLRP3 inflammasome. Immunoblot analysis of NLRP3, ASC, proCaspase-1, Caspase-1 p20, pro-IL-1β, and actin after curdlan **(A)** or CpG stimulation **(B)** for different periods of time, as indicated. **(C)** Capase1 activity was determined in unstimulated cells (NTC), after Curdlan (Curd) and CpG stimulation. In some cases, the stimulation was performed in the presence of the caspase inhibitor YVAD-CMK (50 µM) as indicated. IL-1β ELISA measured in the cell supernatant of NTC, after curdlan stimulation and after curdlan stimulation of cells pretreated with VX-765 and YVAD-CMK **(D)**, 50 mM KCL and 50 µM oxidized ATP (oxATP) **(E)** and 100 µM MCC950 **(F)** as indicated. 200 µg/ml curdlan and 1 µg/ml CpG were used for stimulation. Data are representative of at least three independent experiments. *p < 0.05, **p < 0.01, ***p < 0.0001.

The NLRP3 inflammasome is crucial for the processing of biologically inactive IL-1β (pro-IL-1β) into the mature and biologically active form (IL-1β). It was therefore important for us to determine its role in β-glucan-mediated IL-1β secretion. Protein expression of NLRP3, ASC, caspase-1, and pro-IL-1β as well as caspase-1 activity were therefore assessed and found to increase significantly upon β-glucan stimulation (Figures 2A,C). CpG stimulation did not induce pro-IL-1β, a required step needed for mature IL-1β production (Figure 2B). However, despite the lack of increase in pro-IL-1β, CpG also resulted in activation of the NLRP3 inflammasome (Figures 2B,C).

To further demonstrate that Caspase-1 and NLRP3 were indeed important in β-glucan-mediated IL-1β secretion and since primary human B-lymphocytes are not suitable for lentivirus infection or transfection with other forms of interfering RNA, IL-1β was measured in the cell supernatant of stimulated cells in the presence of different Caspase-1 and NLRP3 inhibitors. As shown in Figures 2D–F, the use of the caspase-1 inhibitors VX765 and YVAD-CMK, KCL, oxATP, and the specific NLRP3 inhibitor, MCC950 (19), resulted in significant decreases of IL-1β secretion, confirming the participation of the canonical NLRP3 inflammasome pathway in β-glucan-mediated IL-1β secretion. Absence of cell toxicity was confirmed for all the inhibitors as shown in Supplement S1 in Supplementary Material.

**Figure 3:**
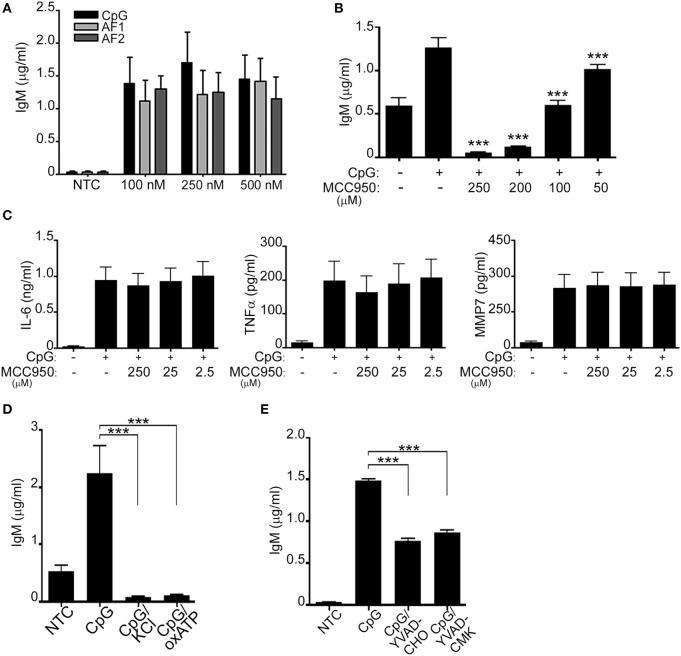
CpG induces NLRP3-mediated antibody production. IgM **(A,B)**, IL-6, TNF-α, and MMP-7 **(C)** were measured by ELISA in the cell supernatant of unstimulated (NTC), CpG, AF1 and AF2 stimulated cells, and CpG stimulated cells in the presence of different concentrations of MCC950 as indicated. Cells were left untreated (NTC) or pre-incubated with 50 mM KCL, 50 µM oxidized ATP (oxATP) **(D)**, and the caspase-1 inhibitors, YVAD-CHO and YVAD-CMK, at a concentration of 50 µM each **(E)** prior to stimulation with CpG as indicated. 1 µg/ml CpG was used unless otherwise specified. Data are representative of at least three donors in each independent experiments (*n* = 3). ***p < 0.0001.

Since prior data suggested that in NLRP3 deficient mice IgM secretion and antibody production are impaired (8, 20–22), we hypothesized that NLRP3 may regulate IgM secretion. We and others have additionally shown that peripheral B-lymphocytes secrete IgM in response to CpG (ODN 2006) (7, 23) (Supplement S2 in Supplementary Material). To better understand the role of NLRP3 activation in CpG-stimulated B-lymphocytes we further investigated if IgM was also triggered in response to AF1 and AF2. As shown in Figure 3A IgM was similarly induced by all forms of CpG tested. Next, we evaluated IgM levels after CpG stimulation in the presence of different concentrations of MCC950. Interestingly, the levels of IgM decreased in the presence of MCC950 in a dose-dependent manner (Figure 3B; Supplement S3A in Supplementary Material). To ensure that this effect was specific to inhibition of the NLRP3 inflammasome, IL-6, MMP-7, and TNFα were also assessed in the presence of MCC950. As shown in Figure 3C, none of the other cytokines and metalloproteases were affected by the presence of the inhibitor further supporting the specific role of the NLRP3 inflammasome in IgM secretion. Additionally, IgM release was also impaired in the presence of KCL, oxATP, and caspase inhibitors (Figures 3D,E; Supplement S3A in Supplementary Material), all well-known agents that affect the functioning of the NLRP3 inflammasome.

### Dectin-1 and Syk-Participate in NLRP3 Activation by β-Glucans While CpG Signaling Activation of NLRP3 Regulates IgM *via* mTOR

Since the inflammasome was activated by both β-glucan and CpG and both are known to signal through very different PRRs, we next explored the specific signaling pathways that lead to IL-1β and IgM secretion upon β-glucan and CpG, respectively.

**Figure 4:**
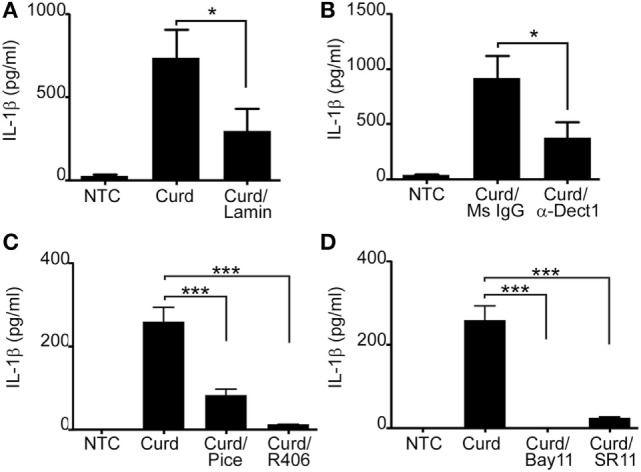
Dectin-1 partially mediates IL-1β secretion and requires SYK and the transcriptions factors AP-1 and NF-κB. IL-1β ELISA measured in the cell supernatant of curdlan (Curd) stimulated cells in the presence or absence of 1 mg/ml Laminarin (Lamin) **(A)**, 5 µg/ml anti-dectin-1 antibody (α-Dect1) or 5 µg/ml isotype control (Ms IgG) **(B)**, 40 µM of the SYK inhibitor, piceatannol (pice) and 5 µM of the SYK inhibitor, R406 **(C)**, and 10 µM of the NF-κB inhibitor, Bay11, and 10 µM of the AP-1 inhibitor, SR11 **(D)**. Data are representative of at least three donors in each independent experiments (*n* = 3). *p < 0.05, ***p < 0.0001.

β-Glucans are known to signal predominantly through the C-lectin receptor Dectin-1 (6, 7, 24, 25) while CpG is the natural ligand for TLR9 (4, 26). Hence, we first explored the contribution of Dectin-1 in β-glucan-induced IL-1β signaling. B-lymphocytes were pre-incubated with either laminarin (a soluble β-glucan known to bind to Dectin-1 acting as a competitive inhibitor) or a specific Dectin-1 blocking antibody prior to β-glucan stimulation. IL-1β levels were significantly reduced in the presence of both, laminarin and the Dectin-1 antibody, confirming the role of Dectin-1 in β-glucan-mediated IL-1β secretion (Figures 4A,B). β-glucan-mediated IL-1β was also dependent on Syk and the transcription factors NF-κB and AP-1 (Figures 4C,D).

**Figure 5:**
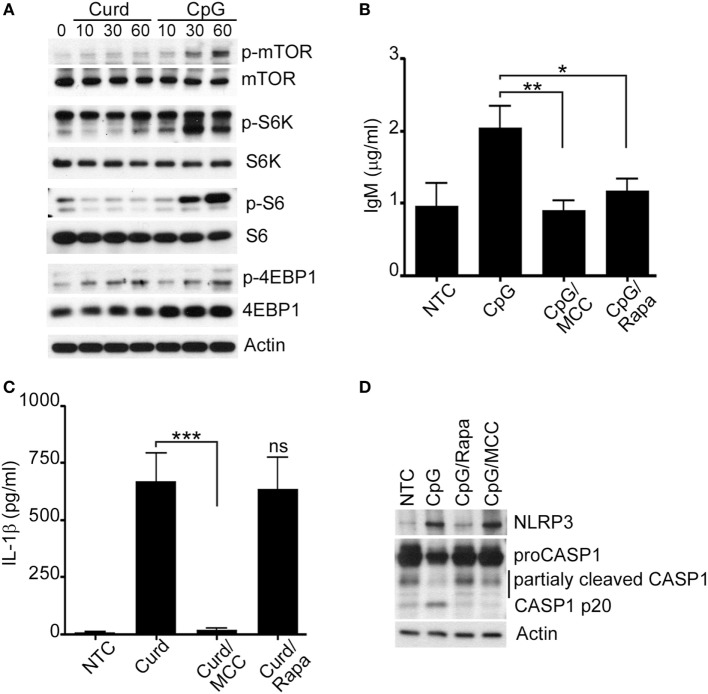
Mammalian target of rapamycin (mTOR) regulates IgM production. **(A)** Immunoblot analysis of p-mTOR, p-S6K, p-S6, p-4EBP1 after curdlan (Curd) or CpG stimulation, for the indicated periods of time. Total mTOR, S6K, S6, 4EBP1 and actin were used as protein control. IgM **(B)** and IL-1β **(C)** ELISA were measured in the cell supernatant of unstimulated cells (NTC), CpG stimulated cells, and CpG stimulated cells in the presence of MCC950 (MCC) and Rapamycin (Rapa) for 24 h. **(D)** Immunoblot analysis of NLRP3, and Caspase-1 in NTC, after CpG stimulation in the presence and absence of MCC950 (MCC) and Rapamycin (Rapa). Actin was used as protein loading control. Cells were stimulated with 200 µg/ml curdlan and 1 µg/ml CpG and pre-incubated with 100 µM of MCC950 and 100 nM Rapamycin. Data are representative of at least three donors in each independent experiments (*n* = 3). *p < 0.05, **p < 0.01, ***p < 0.0001.

TLR9 is the main receptor for CpG motifs found in bacterial and fungal DNA (4, 26) and it is known that stimulation by CpG also involves mTOR (4, 6, 27–29). In contrast, β-glucan stimulation does not seem to require mTOR (6). Thus, to additionally understand the potential involvement of mTOR activation in IgM and IL-1β regulation, phosphorylation of mTOR and other mTOR-related proteins were assessed after cells were stimulated for different period of time with CpG or β-glucan. mTOR and other mTOR-related proteins (S6K, S6, and 4EBP1) tested were phosphorylated upon CpG but not after β-glucan stimulation with the exception of 4EBP1 which seemed to be phosphorylated by both (Figure 5A; Supplement S3B in Supplementary Material). β-glucan activation of 4EBP1 was inhibited in the presence of PD98059, a specific ERK1/2 inhibitor, and not by Rapamycin, a well-known mTOR inhibitor, suggesting that 4EBP1 activation, in β-glucan-stimulated cells, was mTOR-independent and required ERK1/2 (Supplement S4 in Supplementary Material), consistent with prior observations (30).

As our data suggested that NLRP3 regulates IgM *via* mTOR, we measured IgM levels after CpG-stimulation in the presence and absence of Rapamycin. The use of Rapamycin resulted in significant reduction of IgM thus confirming the role of mTOR signaling in IgM regulation (Figure 5B; Supplement S3A in Supplementary Material). The levels of IgM in the presence of Rapamycin were comparably reduced to the levels of IgM after MCC950. In contrast, β-glucan stimulation of IL-1β, while reduced in the presence of MCC950, was not affected by the use of Rapamycin confirming that this is an mTOR-independent process (Figure 5C). Interestingly, Rapamycin inhibition of the NLRP3 inflammasome seemed to affect Caspase-1 activation as shown in Figure 5D. Similar observations were made for MCC950, consistent with prior published observations (31).

### Memory B-Lymphocytes Are the Main Producers of IL-1β and IgM

**Figure 6:**
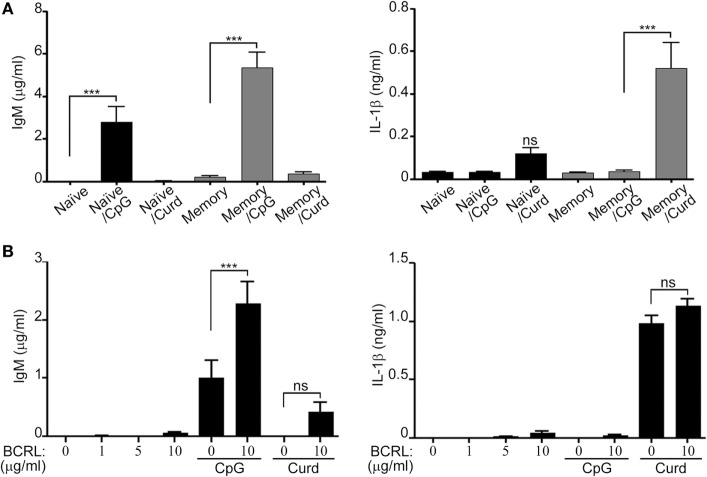
Different levels of IL-1β and IgM secretion by stimulated naïve and memory B-lymphocytes. B-lymphocytes were isolated by negative selection, stained for 30 min with anti-CD20 and anti-CD27 antibodies, and sorted into (CD20^+^CD27^+^ and CD20^+^CD27^−^). Sorted cells were then plated at 2 × 10^6^/ml and stimulated with CpG and curdlan (Curd) **(A)** or with B cell receptor ligand (BCRL) alone or in combination with CpG or curdlan **(B)** as indicated. IgM and IL-1β ELISA were measured in the cell supernatant 72 h later. 200 µg/ml curdlan and 1 µg/ml CpG were used unless otherwise specified. Data are representative of at least three donors in each independent experiment (*n* = 2) ***p < 0.0001, ns, not significant (*p* > 0.05).

Peripheral B-lymphocytes can be divided into naïve and memory cells. In order to further understand the subtype of B-lymphocytes responsible for the secretion of IL-1β and IgM, and since it is clearly established that naïve and memory cells produce different cytokine patterns upon stimulation (32, 33). We isolated B-lymphocytes by negative selection and then sorted them into CD20^+^CD27^−^ (naïve) and CD20^+^CD27^+^ (memory) cells. The two different subtypes of B-lymphocytes were independently stimulated with either β-glucan or CpG. IL-1β and IgM levels in the cell supernatant were then measured by ELISA. As Figure 6A shows, we observed that IL-1β was mostly secreted by memory cells whereas IgM production was greater from the sorted memory cells and significantly higher than naïve cells (*p* < 0.001), but was still seen at significant levels in the naïve cells upon CpG stimulation. Co-stimulation of the BCR receptor resulted in an increase in IgM secretion but it did not have a significant effect on IL-1β secretion (Figure 6B).

## Discussion

In this study, we demonstrate that in human peripheral B-lymphocytes the NLRP3 inflammasome is differentially activated by fungal β-glucan and CpG antigens. Activation of NLRP3 by fungal β-glucan resulted in the cleavage of pro-IL-1β into its active form. While this is a well-known function of the NLRP3 in other cell types such as macrophages and dendritic cells, its role in peripheral human B-lymphocytes was not yet clearly understood. Furthermore, herein we have shown that IL-1β secretion was mediated through the major β-glucan receptor Dectin-1 and required signaling through Syk, consistent with our prior observations of β-glucan signaling in B-lymphocytes and current knowledge in other immune cells (6, 7, 11, 34–36). IL-1β regulation was mediated by the transcription factors NF-κB and AP-1 known to be necessary for the transcription of pro-IL-1β (35, 37). Once pro-IL-1β is formed then subsequent activation of the NLRP3 inflammasome is necessary for the processing of the inactive pro-IL-1β into the active or mature form. Activation of the inflammasome *via* the canonical and non-canonical pathways has been described in other cell types (13, 35, 38). For instance, in dendritic cells, Dectin-1 participates not only in the activation of NF-κB and synthesis of pro-IL-1β by curdlan-activated dendritic cells but also in the production of mature IL-1β through a non-canonical inflammasome that recruits ASC and caspase-8 to the complex CARD-Malt-BCL10 (38). Caspase-8 has also been reported as an effector and regulator molecule of the canonical NLRP3 inflammasome that drives IL-1β production (13). Herein, we demonstrate that stimulation of B-lymphocytes with β-glucan and CpG resulted in the activation of the canonical NLRP3 pathway and involved both ASC and caspase-1 activation. Interestingly, while β-glucan-mediated IL-1β secretion via Dectin-1-Syk-NF-κB/AP-1 pathways and canonical NLRP3 activation, CpG stimulation of B-lymphocytes also resulted in the activation of the canonical NLRP3 inflammasome, but triggered the secretion of IgM and not IL-1β. The lack of IL-1β was likely due to the absence of pro-IL-1β which limited the production of the active form since no substrate was available.

Studies have shown that host immunity against disseminated *candida, aspergillosis*, and *pneumocystis* infection relies on IL-1β to clearly mount an adequate immune response, particularly in early stages of disease (17, 39–41). Impaired IL-1β secretion results in decreased recruitment of neutrophils, macrophages, and lymphocytes to the lungs, affecting the phagocytosis and killing of the fungi (17). IL-1β also acts by activating the release of other inflammatory cytokines, like IL-6 and TNFα as well as inducing Th17 polarization, all important mediators in antifungal defense (42). Furthermore, IL-1β is also involved in the pathogenesis of many inflammatory diseases such as familial mediterranean fever, familial cold-induced auto-inflammatory syndrome, steroid-resistant asthma, and rheumatoid arthritis (43–46). In most of these diseases, blocking IL-1β by monoclonal antibodies or blocking the NLRP3 inflammasome provide symptomatic relief and are currently the standard clinical treatment strategies. Understanding IL-1β regulation in B-lymphocytes offers a new way to potentially develop treatments that will continue to improve the clinical course of patients, not only during the acute infectious process, but potentially by ameliorating these chronic inflammatory conditions as well.

Over the last decade, microbiome studies have clearly demonstrated that the lower respiratory tract is colonized by many organisms and is not sterile as initially assumed. It is very likely that the lung microbiome, similar to the intestinal microbiome, plays an important role in maintaining the well-functioning defense mechanisms of the respiratory mucosa (47). It is also highly probable that bacterial and fungal antigens, through activation of PRRs, help to keep the immune system in a basal activation state that ultimately results in the well-being of the host. However, if this balance is disrupted, and the microbiota loses diversity, the more prevalent microbial antigens through the same PRR would have no regulatory negative feedback resulting in an exaggerated inflammatory response. In the clinical setting, single or paucity fungal colonization in the airway is not uncommon, particularly in immunosuppressed patients. While the clinical significance of this finding is unknown, in the majority of the cases it gets overlooked, as the thought is that it does not result in a clinically significant illness. While this may be true, our observations suggest that fungal antigens are potential triggers of IgM, IL-1β, and other inflammatory cytokines (IL-8, IL-6, and TNFα) that can potentially act as chronic stimuli for B-lymphocytes. Thus, it is important to recognize that fungal activated B-lymphocytes, while they can contribute to host protection against fungal diseases in the right clinical setting, they can also result in a source of chronic inflammation.

**Figure 7:**
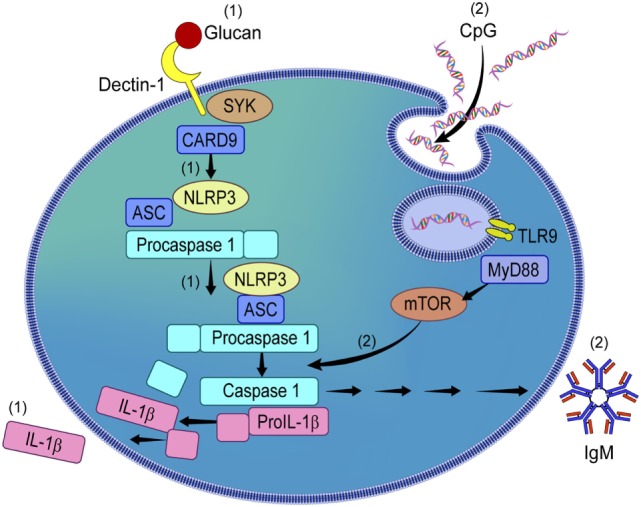
Proposed mechanism of NLRP3 activation by β-glucan and CpG in circulating human B-lymphocytes. β-glucan and CpG activate NLRP3 in peripheral B-lymphocytes. While β-glucan activated B-lymphocytes secrete IL-1β *via* NLRP3 activation **(1)**, CpG does not induce IL-1β but IgM **(2)**. CpG-induced IgM *via* NLRP3 is mammalian target of rapamycin (mTOR)-dependent but not β-glucan-mediated IL-1β secretion.

Contrary to what we observed in β-glucan-stimulated B-lymphocytes, our data also showed that CpG, while activating the NLRP3 inflammasome, did not result in IL-1β secretion, but upregulated IgM secretion *via* the mTOR signaling pathway (see proposed mechanism in Figure 7). The role of NLRP3 in antibody production has been suggested in animal models (8); however it has not been investigated in human B-lymphocytes. Herein, we showed that inhibition of NLRP3 decreased IgM production in a dose-dependent manner while not affecting the secretion of other cytokines such as TNF-α, IL-6, and MMP-7, suggesting that the production of IgM is seemingly controlled by the NLRP3 inflammasome.

The mTOR pathway is ubiquitously expressed in immune cells, including B-lymphocytes (48, 49). Upon activation it regulates important cell processes such as protein translation, cell growth and proliferation (50). mTOR functions as two signaling complexes, mTOR complex 1 (mTOR1) and mTOR complex 2 (mTOR2). Activation of mTOR1 results in phosphorylation of p70-s6K (S6K), S6, and eukaryotic initiation factor 4E-binding protein (4E-BP1) which is important for the regulation of protein synthesis (50). Herein, we found phosphorylation of S6, S6K, and 4EBP-E after CpG stimulation. Furthermore, the use of Rapamycin, a well-described mTOR1 inhibitor, decreased IgM secretion. The use of Rapamycin also affected NLRP3 and caspase-1 activation, therefore impairing the function of NLRP3 inflammasome complex and suggesting that IgM regulation is not only mediated by NLRP3 but also by mTOR. Prior observations in macrophages also suggest that mTOR inhibition suppresses NLRP3 inflammasome activation in macrophages (31). In their study, mTOR regulated HK1-dependent glycolysis was critical for NLRP3 activation. While further investigations are needed in B-lymphocytes, studies to deeply understand the specific mechanism by which mTOR and NLRP3 are controlling antibody regulation in B-lymphocytes studies have shown that mTOR participates in the regulation of high affinity antibodies (51). Furthermore, in animal models, impaired mTOR signaling in follicular and marginal B-lymphocytes stimulated by LPS show a marked reduction of IgM and IgG (50, 52). That mTOR is important for germinal center formation is known (48) however, little is known about the unique function of mTOR in circulating human B-lymphocytes. Here, we have demonstrated the participation of mTOR in NLRP3 function and regulation of IgM. Interestingly, while β-glucans are able to trigger different cytokines and chemokines, they fail to induce IgM in human peripheral B-lymphocytes (7). These data differ from prior studies in murine models in which curdlan stimulation seemed to induce IgM secretion (8). While the differences seen between the two experimental models could be explained by the differences in the experimental methodology and the lack of β-glucan to induce proliferation in the human cells; herein, we have shown the inability of curdlan to trigger mTOR activation, a step found to be important for IgM secretion. Our observations are novel and raise awareness of the role of NLRP3 as regulator not only of IL-1β and IL-18 but also of IgM. Interestingly, despite NLRP3 activation, IL-18 was not induced by β-glucan or CpG in peripheral B-lymphocytes (7).

Beyond their antibody-producing role, B-lymphocytes can affect the local immune environment through the release of cytokines and other proteins; however, how they modulate and contribute to the orchestration of the innate immune system is not clearly understood. Our group has recently shown that activated B-lymphocytes participate in the recruitment of neutrophils by releasing IL-8 and indirectly by contributing to the release of syndecan-4 *via* MMP-7 (6, 7). Syndecan proteins are expressed on the cell membrane and can be shed into the extracellular space by metalloproteases (53). Upon shedding they are free to bind to different cytokines participating in the regulation of cytokine influx and neutrophil recruitment (54–56). Herein, we further demonstrated that B-lymphocytes also contribute to the pool of IL-1β, a pro-inflammatory cytokine very important for antimicrobial host defense (42) and to the secretion of IgM antibodies. Naïve and memory B-lymphocytes play different roles in the regulation of the immune response by releasing different cytokine profiles (2). Here, we investigated the specific B-lymphocyte subtype responsible for IL-1β and IgM secretion and contrary to what we have observed with MMP-7, which was mostly released by naïve cells, memory cells were found to be the main source for both IL-1β and IgM (6). While the exact mechanisms by which different B-lymphocyte subtypes seem to preferentially secrete a specific cytokine milieu is not totally understood, these patterns are not fixed and can be influenced by the circumstances that surround their activation such as presence of T-lymphocytes and other inflammatory cells, by the integration of different signals from multiple receptors and by the proportion of the different B-lymphocytes compartments (2, 33, 57). These are important observations with therapeutic implications as B-lymphocyte reconstitution after Rituximab (B-lymphocyte depleted agent) treatment favors naïve B-lymphocytes (33). Increased numbers of naïve B-lymphocytes, based on our observations, will result in a decrease of IL-1β and IgM levels potentially contributing to a less inflammatory milieu. How these changes may affect antimicrobial and anti-inflammatory host response need to be further investigated.

In summary, this study identified that the NLRP3 inflammasome is essential for two independent processes, pro-inflammatory cytokine secretion and antibody regulation. Whether NLRP3 activation resulted in cytokine or antibody regulation depended on the stimulating antigen with IL-1β secretion being triggered by fungal β-glucans and IgM by CpG. NLRP3 regulation was also differently regulated upon β-glucan or CpG antigens and was mTOR-independent when stimulated by β-glucans but mTOR-dependent upon CpG stimulation. The new role of the NLRP3 inflammasome and the mTOR pathway as regulators of IL-1β and IgM in activated B-lymphocytes studied here offers a potential new strategy to treat autoimmune disease in which IL-1β and pathogenic IgM antibodies may play a role, particularly if triggered by infectious agents. Further investigations are therefore needed to clarify the potential benefits of mTOR and NLRP3 inhibitors in these clinical settings.

## Author Contributions

Concept and design; data acquisition; and data interpretation/analysis: EC, MA, HD, and VV; manuscript drafting: EC, MA, and VV; manuscript review: all authors.

## Conflict of Interest Statement

The authors declare that the research was conducted in the absence of any commercial or financial relationships that could be construed as a potential conflict of interest.
