# Supplementary material for: Canonical Stimulation of the NLRP3 Inflammasome by Fungal Antigens Links Innate and Adaptive B-Lymphocyte Responses by Modulating IL-1β and IgM Production
Source: Front Immunol. 2017 Nov 9;8:1504. doi: 10.3389/fimmu.2017.01504 (PMC5684107; doi:10.3389/fimmu.2017.01504)

Canonical Stimulation of the NLRP3 Inflammasome by Fungal Antigens Links Innate and Adaptive B-lymphocyte Responses by Modulating IL-1β and IgM Production.

Mohamed F. Ali[*]

Harika Dasari[*]

Virginia P. Van Keulen[*]

Eva M. Carmona[*,#,†]

*From the Thoracic Diseases Research Unit and the #Division of Pulmonary Critical Care and Internal Medicine, Department of Medicine Mayo Clinic and Foundation, Rochester, Minnesota 55905

†Corresponding Author: Eva M Carmona, MD, PhD, 8-48 Stabile Building, Mayo Clinic, Rochester, Minnesota, 55905, Phone: (507) 284-4162, FAX (507) 284-4521, E-mail: carmona.eva@mayo.edu

Supplement 1. Cell viability after different specific inhibitors. XTT was determined in untreated cells (NTC) or after the use of VX765, YVAD-CMK, MCC950, oxATP and KCL at the concentrations indicated. Data are representative of at least two independent experiments.

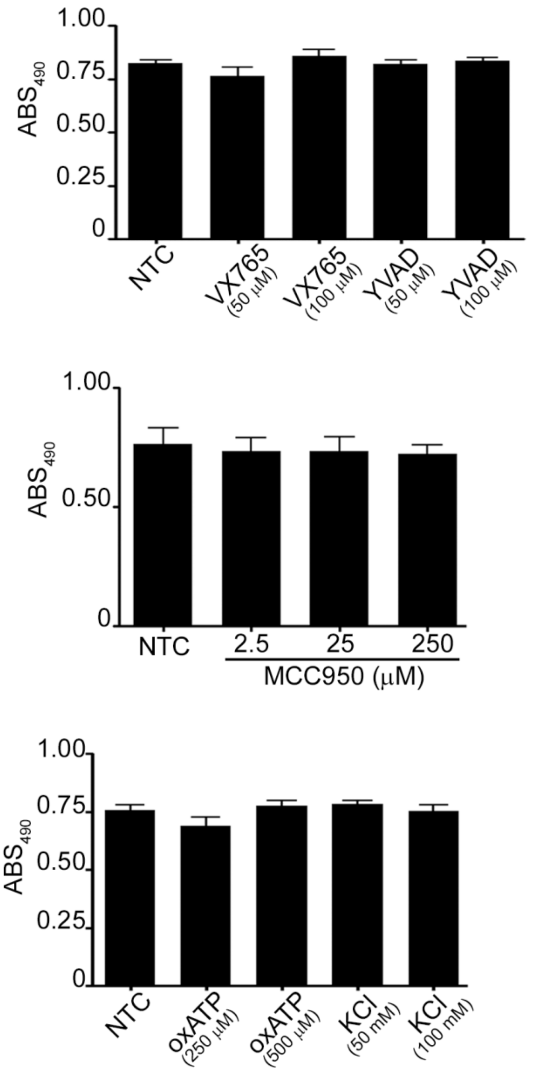

Suppment 2. IgM release after CpG stimulation. IgM was measured by ELISA in the cell supernantant of B-lymphocytes after different concentrations of CpG or after different periods of time. Data are representative of at least two independent experiments.

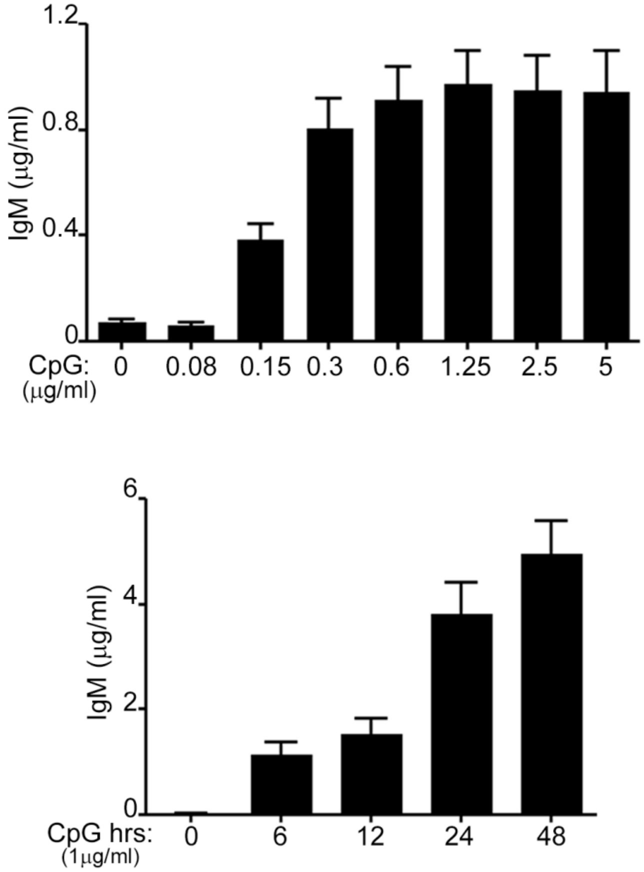

Supplement 3. Fungal CpG from *Aspergillus fumigatus* stimulate IgM via NLRP3 and mTOR. **(A)** IgM was measured by ELISA in the cell supernatant of unstimulated (NTC) and AF1 stimulated cells in the presence of 50μM oxATP, 50mM KCL, 50μM YVAD-CHO , 50μM YVAD-CMK, 100 μM of MCC950 and 100nM Rapamycin. **(B)** Immunoblot analysis of p-mTOR and total mTOR after stimulation with CpG, AF1 and AF2 as indicated. CpG, AF1 and AF2 were used at a concentration of 1μg/ml. Data are representative of at least three donors of three independent experiments. ***p<0.0001.

A]

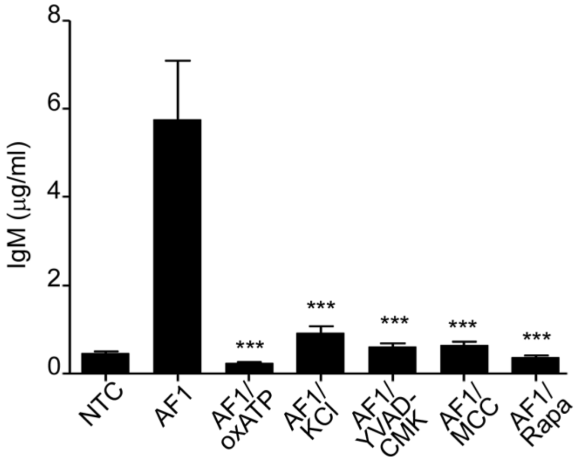

B]

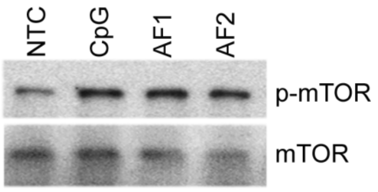

Supplement 4. 4EBP1 activation *via* ERK 1/2. Immunoblot analysis of p-4EBP1, p-ERK and p-mTOR in nonstimulated cells (NTC) and after curdlan (Curd) stimulation for 30 min. Some cells were preincubated with the ERK 1/2 inhibitor, PD98059 (PD), and Rapamycin (Rapa) as indicated. Total 4EBP1, ERK 1/2 and mTOR were used as loading control. PD was used at a concentration of 10μM and Rapamycin at 100nM. Data are representative of at least three independent experiments with different donor preparations.

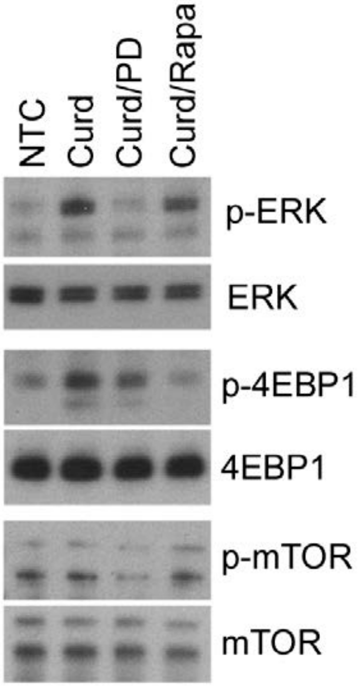

Supplement: Supplementary file 1 [file Presentation_1.pdf]
